# Draft Genome Sequences of Two Uncultured *Armatimonadetes* Associated with a *Microcystis* sp. (*Cyanobacteria*) Isolate

**DOI:** 10.1128/genomeA.00717-17

**Published:** 2017-10-05

**Authors:** Jason N. Woodhouse, A. Katharina Makower, Hans-Peter Grossart, Elke Dittmann

**Affiliations:** a Department of Experimental Limnology, Leibniz-Institute of Freshwater Ecology and Inland Fisheries, Stechlin, Germany; b School of Biotechnology and Biomolecular Sciences, University of New South Wales, Sydney, Australia; c Department of Microbiology, University of Potsdam, Potsdam, Germany

## Abstract

Two genome sequences of the phylum *Armatimonadetes*, derived from terrestrial environments, have been previously described. Here, two additional *Armatimonadetes* genome sequences were obtained via single-molecule real-time (SMRT) sequencing of an enrichment culture of the bloom-forming cyanobacterium *Microcystis* sp. isolated from a eutrophic lake (Brandenburg, Germany). The genomes are most closely affiliated with the class *Fimbriimonadales*, although they are smaller than the 5.6-Mbp type strain genome.

## GENOME ANNOUNCEMENT

Members of the phylum *Armatimonadetes* occur in diverse environments, e.g., on human skin, in temperate soils, bioreactors, and freshwater lakes (1). To date, three strains have been isolated, *Armatimonas rosea* YO-36^T^, from the roots of the aquatic plant *Phragmites australis* (2), *Chthonomonas calidirosea* T49^T^, from geothermal soil (3), and *Fimbriimonas ginsengisoli* Gsoil 348^T^, from soil associated with ginseng cultivars (4). The three isolates exhibit between 77 and 81% pairwise nucleotide similarity across the 16S rRNA gene. Genome analyses of *F. ginsengisoli* (5) and *C. calidirosea* (6) revealed differences in genome size, GC content, protein homology, and gene synteny, but similarities exist with regard to the utilization of complex sugars, ammonium assimilation, and the uptake of branched-chain amino acids to supplement nutrient requirements (5).

In 2011, four colony-forming *Microcystis* strains from Lake Zernsee, Potsdam, Germany, were grown in BG-11 medium (7) at 25°C and continuous illumination (16 µmol photons m^−2^ s^−1^). Of these, one strain, *Microcystis* sp. UP-HVL2, was sequenced using the RSII platform (Pacific Biosciences) at GATC Biotech (Constance, Germany) and assembled using the SMRT Portal *de novo* workflow. Two *Armatimonadetes* genomes, those of Uphvl-Ar1 and Uphvl-Ar2, were obtained from tetranucleotide binning of the *Microcystis* sp. UP-HVL2 genome using MetaWatt version 3.5.3 (8), with default parameters. Genome completeness and contamination estimated of the genomes as assessed using CheckM (9) were 94.91% and 0.93% for Uphvl-Ar1 and 89.81% and 0.93% for Uphvl-Ar2, respectively.

Annotation was performed using the NCBI Prokaryotic Genome Annotation Pipeline. Uphvl-Ar1 comprised a single 2.99-Mbp contig, 3,131 gene features, and a GC content of 52.5%. Uphvl-Ar2 comprised a single 2.6-Mbp contig, 2,644 gene features, and a GC content of 62.1%. Uphvl-Ar1 and Uphvl-Ar2, consistent with Gsoil348^T^, lacked a complete 16S-23S-5S rRNA operon. In both instances, a 0.8- to 0.84-Mbp region separated the 16S rRNA gene from the 23S-5S region. The 16S rRNA genes of Uphvl-Ar1 and Uphvl-Ar2 exhibited 90% homology with one another. The 16S rRNA genes of Uphvl-Ar1 exhibited 91% sequence homology with those from uncultured *Armatimonadetes* from bioreactors (GenBank accession numbers KT182498, EF648093, KJ783172, and HQ864199), whereas Uphvl-Ar2 exhibited 99% homology with those from uncultured *Armatimonadetes* from freshwater (accession numbers HQ860522, GU305825, DQ501336, and FJ662688). The SINA classifier placed both strains within the family *Fimbriimonadaceae*, with ~87% identity homology with reference strains. In contrast to the terrestrial *F. ginsengisoli*, the aquatic Uphvl-Ar1 and Uphvl-Ar2 are both likely motile, possessing complete flagellum biosynthetic pathways and numerous chemotaxis receptors for simple sugars and amino acids. Uphvl-Ar1 and Uphvl-Ar2 lack the ability to take up ammonium or nitrate, instead relying on the uptake and hydrolysis of amino acids, including glutamine, to meet their nitrogen requirements.

### Accession number(s).

The sequences of Uphvl-Ar1 and Uphvl-Ar2 have been deposited in GenBank under the accession numbers CP021423 and CP021424, respectively.
